# Bcr-TMP, a Novel Nanomolar-Active Compound That Exhibits Both MYB- and Microtubule-Inhibitory Activity

**DOI:** 10.3390/cancers14010043

**Published:** 2021-12-23

**Authors:** Maria V. Yusenko, Abhiruchi Biyanee, Daria Frank, Leonhard H. F. Köhler, Mattias K. Andersson, Cyrus Khandanpour, Rainer Schobert, Göran Stenman, Bernhard Biersack, Karl-Heinz Klempnauer

**Affiliations:** 1 Institute for Biochemistry, Westfälische-Wilhelms-Universität, 48149 Munster, Germany; maria.yusenko@uni-muenster.de (M.V.Y.); abhiruchi.biyanee@uni-muenster.de (A.B.); 2 Department of Medicine A, Hematology and Oncology, University Hospital, Westfälische-Wilhelms-Universität, 48149 Munster, Germany; Daria.Frank@ukmuenster.de (D.F.); Cyrus.Khandanpour@ukmuenster.de (C.K.); 3 Organic Chemistry Laboratory, Universität Bayreuth, 95440 Bayreuth, Germany; Leonhard.Koehler@uni-bayreuth.de (L.H.F.K.); Rainer.Schobert@uni-bayreuth.de (R.S.); bernhard.biersack@uni-bayreuth.de (B.B.); 4 Sahlgrenska Center for Cancer Research, Department of Pathology, University of Gothenburg, 41345 Gothenburg, Sweden; mattias.andersson@llcr.med.gu.se (M.K.A.); goran.stenman@llcr.med.gu.se (G.S.)

**Keywords:** MYB, inhibitor, acute myeloid leukemia, adenoid cystic carcinoma, microtubule disruption

## Abstract

**Simple Summary:**

Recent work has identified the transcription regulator MYB as an interesting therapeutic target for the treatment of certain leukemias and other cancers that are dependent on deregulated MYB activity, such as acute myeloid leukemia (AML) and adenoid cystic carcinoma (ACC). Here we report the identification and characterization of 2-amino-4-(3,4,5-trimethoxyphenyl)-4*H*-naphtho[1,2-*b*]pyran-3-carbonitrile (Bcr-TMP), a novel highly active MYB inhibitory compound. We show that nanomolar concentrations of Bcr-TMP are sufficient to down-regulate the expression of MYB target genes and induce both cell-death and differentiation in AML cell lines. Importantly, Bcr-TMP also and exerts stronger anti-proliferative effects on MYB-addicted primary AML cells and patient-derived ACC cells than on their non-oncogenic counterparts. Preliminary work shows that Bcr-TMP acts through p300, a protein interacting with MYB and stimulating its activity. Interestingly, Bcr-TMP has an additional activity as an anti-microtubule agent. Overall, Bcr-TMP is an interesting compound that warrants further research to understand its mechanism of action and its therapeutic potential for MYB-dependent malignancies.

**Abstract:**

Studies of the role of MYB in human malignancies have highlighted MYB as a potential drug target for acute myeloid leukemia (AML) and adenoid cystic carcinoma (ACC). Here, we present the initial characterization of 2-amino-4-(3,4,5-trimethoxyphenyl)-4*H*-naphtho[1,2-*b*]pyran-3-carbonitrile (Bcr-TMP), a nanomolar-active MYB-inhibitory compound identified in a screen for novel MYB inhibitors. Bcr-TMP affects MYB function in a dual manner by inducing its degradation and suppressing its transactivation potential by disrupting its cooperation with co-activator p300. Bcr-TMP also interferes with the p300-dependent stimulation of C/EBPβ, a transcription factor co-operating with MYB in myeloid cells, indicating that Bcr-TMP is a p300-inhibitor. Bcr-TMP reduces the viability of AML cell lines at nanomolar concentrations and induces cell-death and expression of myeloid differentiation markers. It also down-regulates the expression of MYB target genes and exerts stronger anti-proliferative effects on MYB-addicted primary murine AML cells and patient-derived ACC cells than on their non-oncogenic counterparts. Surprisingly, we observed that Bcr-TMP also has microtubule-disrupting activity, pointing to a possible link between MYB-activity and microtubule stability. Overall, Bcr-TMP is a highly potent multifunctional MYB-inhibitory agent that warrants further investigation of its therapeutic potential and mechanism(s) of action.

## 1. Introduction

MYB is the founding member of the MYB family of oncogenic transcription factors, which consists of MYB, MYBL1, and MYBL2 in vertebrate species [1,2,3]. MYB is most highly expressed in hematopoietic progenitor cells where it acts as a master regulator of gene expression programs to control the development and homeostasis of the hematopoietic system. Genome-wide studies have identified a broad spectrum of MYB-regulated genes with critical roles in important biological processes, including cell proliferation and differentiation [4,5]. The MYB protein features an N-terminal DNA-binding domain recognizing a short nucleotide binding motif [6], a centrally-located transactivation domain that stimulates the transcription of target genes [7,8], and C-terminal regulatory sequences that are post-translationally modified by phosphorylation, acetylation, and sumo-conjugation [9,10,11,12]. As a transcription activator, MYB is highly dependent on co-operation with the co-activator p300/CBP, which binds via its KIX-domain to a conserved LXXLL-motif located in the MYB transactivation domain [13,14,15]. Mutations causing to amino acid replacements within or immediately adjacent to the LXXLL-motif, such as L302A or M303V, disrupt the interaction with p300 and weaken the transactivation potential of MYB [16,17,18]. Several studies have shown that the oncogenic activity of MYB is linked to its activity as a transcriptional activator, indicating that its oncogenic potential is due to the deregulation of specific target genes.

Besides its role in normal cells, MYB (protein) has been implicated in human neoplasia. Genomic rearrangements of human *MYB (gene)* and mutations that create de novo MYB binding sites in transcriptional control regions of the *TAL1* and *LMO2* oncogenes have been detected in acute lymphoid leukemia, indicating that MYB plays a causal role in the development of these leukemias [19,20,21,22,23]. In acute myeloid leukemia (AML), *MYB* rearrangements are rare; however, AML cells are often addicted to high levels of MYB, making them more vulnerable to MYB inhibition than their normal counterparts [24,25,26]. MYB has also been implicated in certain non-hematopoietic tumors, such as breast and colon cancer [27,28,29,30], adenoid cystic carcinoma (ACC) [31], and diffuse low-grade pediatric gliomas [32,33]. In ACC, recurrent translocations fuse *MYB* with the *NFIB* gene in a high percentage of cases, leading to the expression of oncogenic MYB-NFIB fusion proteins. Similarly, translocations fusing *MYB* with various other loci occur frequently in low-grade pediatric gliomas.

The involvement of MYB in human malignancies has stimulated the interest in MYB as a potential drug target [34,35]. Although transcription factors are traditionally considered as undruggable, several studies have pioneered targeting of MYB by low-molecular weight compounds and have demonstrated that MYB is a druggable transcription factor. Importantly, using in vivo mouse models and patient-derived AML and ACC cells these studies have provided evidence that MYB inhibition may be effective against AML and ACC [36,37,38,39,40,41,42]. Pharmacological inhibition of MYB may therefore have potential as a novel therapeutic strategy against MYB-dependent malignancies.

We have screened chemical compound libraries for low molecular weight MYB inhibitors, using a previously established MYB reporter cell line [42,43,44]. Here, we present the initial characterization of 2-amino-4-(3,4,5-trimethoxyphenyl)-4*H*-naphtho[1,2-*b*]pyran-3-carbonitrile (Bcr-TMP), a novel and highly active MYB-inhibitory compound.

## 2. Materials and Methods

### 2.1. Cells

HEK293T, HepG2, U2OS, HeLa, RPE, MCF7, and 518A2 are non-hematopoietic adherent human cell lines. NB4, HL60, U937, THP1, OCI-AML3, U937, Kasumi1, KG-1, MV-4-11, K562, and Jurkat are human leukemia cell lines. All cell lines were originally obtained from ATCC and are free of mycoplasma contamination. HL60 cells expressing a C-terminally truncated MYB (MYB-CT3) and control HL60 cells were generated by lentiviral infection as described previously [43,45]. AML cells were grown in RPMI1640 medium supplemented with 10% FCS. Adherent human cell lines were grown in DMEM plus 10% FCS. Patient-derived ACC cells were cultured as described [39].

### 2.2. Library Screening and Compounds

A screen of compound libraries covering approximately 62,000 chemical compounds was performed by the screening facility of the FMP (Berlin), using the HEK-MYB-Luc and HEK-Luc reporter cell lines described before [43]. HEK-MYB-Luc cells allow doxycycline-inducible MYB expression and harbor a MYB-dependent luciferase reporter plasmid. Compounds were initially screened at a concentration of 10 μM, using 384-well plates containing doxycycline-pretreated HEK-MYB-Luc cells. After 24 h of incubation, luciferase activities were analyzed with the Steady-Glo luciferase kit (Promega), using a TECAN microplate reader. Data was analyzed based on the Z score (the number of standard deviations a measured signal intensity is above the mean). Selected compounds were first re-tested at 10 μM concentration in triplicates in HEK-MYB-Luc and HEK-Luc cells. Candidate compounds were then subjected to IC_50_ determinations at concentrations between 20 and 0.01 μM. 2-amino-4-(3,4,5-trimethoxyphenyl)-4*H*-naphtho[1,2-*b*]pyran-3-carbonitrile (Bcr-TMP), and related compounds 2-amino-4-(3,4-dimethoxyphenyl)-4*H*-benzo[*h*]chromene-3-carbonitrile (Bcr-DMP) and 2-amino-4-(4-methoxyphenyl)-4*H*-benzo[*h*]chromene-3-carbonitrile (Bcr-MMP) were obtained from Vitas-M. Laboratory. 5-(3,4,5-Trimethoxyphenyl)-5,11-dihydro-1*H*-indeno[2′,1′:5,6]pyrido-[2,3-*d*]pyrimidine-2,4,6(3*H*)-trione (IPU-TMP), LY290181, and 4-(3,4,5-trimethoxyphenyl)-2-(1*H*-pyrrol-1-yl)-4*H*-benzo[*h*]chromene-3-carbonitrile (PyrBcr-TMP) were synthesized by the authors (unpublished). Combretastatin A4 was obtained from Sigma-Aldrich. Finally, 10 mM stock solutions were prepared in DMSO and stored at −70 °C.

### 2.3. Expression Vectors and Transfections

Expression vectors for human MYB, MYB-2KR, MYB-CT3, and p300 have been described before [37,46]. M303V and L302A mutants of MYB-CT3 were generated by site-directed mutagenesis. The MYB-inducible luciferase reporter plasmids pGL4-5xMRE(GG)-Myc (containing 5 tandem copies of a MYB binding site upstream of the Myc core promoter) and pmim3mim-Luc (containing mim1 enhancer and promoter sequences) have been described before [47,48]. HEK293T cells were transfected by calcium-phosphate co-precipitation. Luciferase assays were performed as described before [49].

### 2.4. Electrophoretic Mobility Shift Assays (EMSA)

EMSA was performed with a synthetic double-stranded oligonucleotide that contains a high affinity MYB binding site [48]. For preparation of nuclear extracts, we used non-transfected HEK293T cells or HEK293T that had been transfected with MYB-CT3 expression vector and were incubated for 16 h in the absence or presence of different concentrations of Bcr-TMP.

### 2.5. Quantitative Real-Time PCR

RT-PCR analysis was performed as described before [42]. All experiments were conducted with at least three biological replicates and the following primers:

MYB: 5′-GAAGGTCGAACAGGAAGGTTATCT-3′ and 5′-GTAACGCTACAGGGTATGGAACA-3′; MYC: 5′-GCCGATCAGCTGGAGATGA-3′ and 5′-GTCGTCAGGATCGCAGATGAAG-3′; KIT: 5′-TGATTTTCCTGGATGGATGG-3′ and 5′-TGGGATTTTCTCTGCGTTCT-3′; GFI1: 5′-GCTCGGAGTTTGAGGACTTC-3′ and 5′-ATGGGCACATTGACTTCTCC-3′; ACTB: 5′-AGAGCTACGAGCTGCCTGAC-3′ and 5′-AGCACTGTGTTGGCGTACAG-3′.

### 2.6. Flow Cytometry

Cells were cultured for 2 days in RPMI 1640 medium supplemented with Bcr-TMP at the desired concentration or were cultured without compound, followed by analysis in a FC 500 flow cytometer (Beckman Coulter). fCD11b and CD14 were detected with PE/Cy7-labeled anti-human CD11b (ICRF44, BioLegend) and FITC-labeled anti-human CD14 antibodies (63D3, BioLegend). To quantify apoptotic and necrotic cells, double-staining with FITC-annexin-V (BioLegend) and propidium iodide (PI) was performed. Stained cells were analyzed with the CXP software (Beckman Coulter).

### 2.7. Proliferation and Apoptosis Assays

Cell viability was determined by MTT assays. After incubating the cells for 24 h with Bcr-TMP, MTT solution (Millipore Corp., Bedford, MA, USA) was added and incubated for 4 h. The insoluble formazan product was then dissolved in DMSO and the absorbance was measured at 570 nm with a microplate photometer (MPP 4008, Mikrotek, Overath, Germany). Proliferation assays of ACC cells were carried out as follows. Approximately 4000 cells were seeded per well in black-walled 96-well plates (BD, Franklin Lakes, NJ, USA) and then treated with desired concentrations of Bcr-TMP or DMSO as control; 72 h later the cells were assayed with Alamar blue reagent (Thermo Fisher Scientific, Waltham, MA, USA) according to the manufacturer’s instructions. Apoptosis assays were carried out using approximately 8000 ACC cells per well of a white-walled 96-well plate (BD) and subsequently treated for 24 h with DMSO or Bcr-TMP. Apoptosis was then assayed with the Caspase-Glo 3/7 reagent (Promega, Madison, WI, USA).

### 2.8. Western Blotting

The following antibodies were used to analyze protein expression by Western blotting: anti-MYB (5E11, [50]), anti-β-actin (Sigma-Aldrich, Taufkirchen, Germany, AC-15), anti-C/EBPβ (Santa Cruz Biotechnology, Heidelberg, Germny, sc-7962), anti-p300 (Millipore, Bedford, MA, USA, RW128), anti-GFP (Roche Diagnostics, Mannheim, Germany, clones 7.1 and 13.1), anti-HA (BioLegend, San Diego, CA, USA, 16B12), anti-acetyl-Lysine (Cell Signaling Technology, Frankfurt, Germany, Ac-K-103), and anti-H3K27ac (Abcam, Cambridge, UK, ab4729).

### 2.9. Tubulin Polymerization Assay

In a black 96-well half-area clear bottom plate 50 µL of Brinkley’s Buffer 80 (BRB80: 400 mM PIPES, 5 mM MgCl_2_, 5 mM EGTA, pH 6.8) containing 20% glycerol and 2 mM GTP was pipetted. Bcr-TMP, combretastatin A4 or solvent (DMSO) were added to the wells to reach a concentration of 5 mM. To start the polymerization reaction 50 µL porcine brain tubulin (5 mg/mL in BRB80) was added and the plate was immediately placed in the microplate reader (Tecan infinite F200). The optical density was measured at 37 °C by recording the absorption at 340 nm for 100 min.

### 2.10. Immunofluorescence Staining of Actin and Tubulin Cytoskeleton

518A2 melanoma cells (1 × 10^5^ cells/mL, 0.5 mL/well) were seeded on coverslips in 24-well cell culture plates and incubated for 24 h under cell culture conditions (37 °C, 5% CO_2_, 95% humidity). After adding 10 and 25 nM of Bcr-TMP or combretastatin A4 or the vehicle DMSO, the cells were incubated for 24 h (37 °C, 5% CO_2_, 95% humidity). The cells were washed with cytoskeletal buffer (100 mM PIPES, 3 mM MgCl_2_, 138 mM KCl, 2 mM EGTA, 300 mM sucrose, pH 6.8), fixed and permeabilized in 3.7% formaldehyde and 0.2% Triton X-100 in cytoskeletal buffer for 5 min at RT. As additional fixation step, the cells were incubated with ice-cold EtOH for 10 s and rehydrated in PBS. After blocking with 1% BSA in PBS for 30 min the cells were treated 2 h with a primary antibody against alpha-tubulin (anti-alpha-tubulin, mouse monoclonal antibody, Invitrogen/Life Technologies). The cells were then washed three times with PBS, followed by a 1 h incubation with AlexaFluor^®^-546 conjugated secondary antibodies (goat anti-mouse IgG-AF-546, Invitrogen). Actin and nuclei staining was performed with Acti-Stain 488 phallodin (Cytoskeleton, Inc., Denver, CO, USA) and DAPI (1 µg/mL) for 1 h in the dark. Finally, the cells were washed three times with PBS and the coverslips were embedded in ProLong^TM^ Glass Antifade Mountant. Nuclei, actin filaments and microtubules were documented by confocal microscopy (Leica Confocal TCS SP5).

### 2.11. Statistical Analysis

Experiments subjected to statistical analysis were repeated at least three times. Independent replicates were included in each experiment. Data are shown as mean with standard deviation. Statistical differences between groups were calculated by the two-tailed Student’s *t*-test or by one-way ANOVA. Values of *p* < 0.05 were considered as statistically significant.

## 3. Results

### 3.1. Identification of Bcr-TMP as a Highly Active MYB-Inhibitory Compound

**Figure 1:**
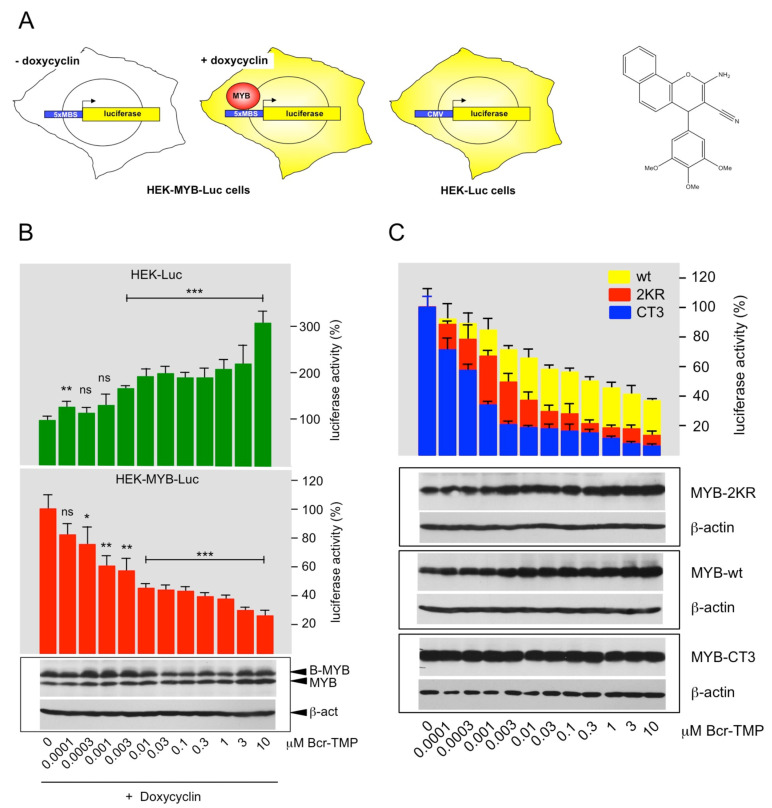
Identification of a highly active MYB-inhibitory compound: (**A**) HEK-MYB-Luc cells carry a MYB-inducible luciferase reporter gene and a doxycycline-inducible expression system for human MYB-2KR. HEK-Luc cells carry a constitutively active luciferase expression vector. The structure of Bcr-TMP is shown on the right. (**B**) HEK-Luc and HEK-MYB-Luc cells were treated for 16 h with doxycycline and the indicated concentrations of Bcr-TMP. Bars show the average luciferase activity of the cells normalized to the luciferase activity of cells treated only with doxycycline. The bottom panels show the expression of MYB, B-MYB (which cross-reacts with the anti-MYB antibody) and β-actin determined by Western blotting. Asterisks indicate statistical significance (ns: non-specific; * *p* < 0.05; ** *p* < 0.01; *** *p* < 0.001; Student’s *t*-test). (**C**) HEK293T cells were transiently transfected with the MYB-responsive reporter plasmid pGL4-5xMRE(GG)-Myc and expression vectors for wt MYB, MYB-2KR and MYB-CT3. Transfected cells were distributed in identical aliquots into microtiter plates and treated for 16 h with the indicated compound concentrations. Bars show the average luciferase activity of the cells normalized to untreated cells. The bottom panels show the expression of MYB, MYB-2KR, MYB-CT3, and β-actin under the different conditions. The uncropped Western blots have been shown in Figure S1.

The MYB reporter cell line HEK-MYB-Luc [43] was used to screen chemical libraries containing approximately 62,000 small molecules, resulting in the identification of 2-Amino-4-(3,4,5-trimethoxyphenyl)-4*H*-benzo[*h*]chromene-3-carbonitrile as a highly active MYB-inhibitory compound. The screening system, the chemical structure of the compound (for simplicity referred to as Bcr-TMP, for “benzochromene-trimethoxyphenyl”) and its effect on the luciferase activity in HEK-MYB-Luc and HEK-Luc cells are illustrated in Figure 1A,B. A comparison of the effects of Bcr-TMP on the luciferase activity of HEK293T-cells transiently transfected with a MYB-dependent reporter plasmid and expression vectors for wt MYB, MYB-2KR (the activated MYB construct expressed in the HEK-MYB-Luc cells) and MYB-CT3 (a C-terminally truncated MYB construct) is shown in Figure 1C. The activity of all three MYB constructs was suppressed by Bcr-TMP; however, wild-type MYB was slightly less sensitive than the other MYB versions. MYB expression remained constant or slightly increased with higher concentration of Bcr-TMP.

### 3.2. Bcr-TMP Decreases the Activity of the MYB Transactivation Domain

**Figure 2:**
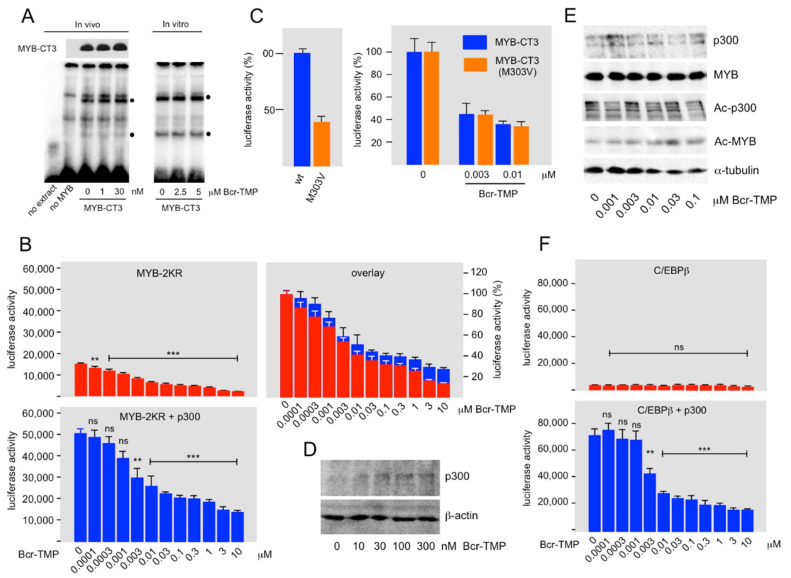
Bcr-TMP suppresses MYB activity in a p300-dependent manner. (**A**) EMSA assay with nuclear extracts from HEK293T cells expressing MYB-CT3, as marked below the lanes, and cultivated for 16 h with the indicated concentrations of Bcr-TMP. Nuclear extract from un-transfected cells was used as control. A Western blot showing MYB-CT3 expression in aliquots of the nuclear extracts is presented at the top. Binding assays were performed with a radiolabeled oligonucleotide containing a consensus MYB binding site. MYB-specific protein-DNA-complexes are labeled by black dots. In addition to using nuclear extracts from cells treated with Bcr-TMP in vivo, EMSA assays were performed by adding compounds directly to the in vitro binding reactions (last three lanes). (**B**) HEK293T cells were transiently transfected with the MYB-dependent reporter plasmid pGL4-5xMRE(GG)-Myc and expression vectors for MYB-2KR and p300, as shown at the top. Transfected cells were treated with Bcr-TMP for 16 h and subjected to luciferase assays. The columns show the absolute luciferase values in arbitrary numbers and the panel on the right (overlay) shows the luciferase activity normalized to that of untreated cells. (**C**) The left panel shows the luciferase activity of HEK293T cells transfected with reporter plasmid pGL4-5xMRE(GG)-Myc and expression vectors for MYB-CT3 and MYB-CT3(M303V). The right panel shows the luciferase activity of HEK293T cells transfected in the same manner and treated without or with the indicated concentrations of Bcr-TMP for 16 h before harvesting and measuring luciferase activities. The difference in activity between MYB-CT3 and the M303V mutant was compensated by expressing the luciferase activities as percent of the activity of the untreated cells. (**D**) Expression of endogenous p300 in HEK293T cells treated for 16 h with or without Bcr-TMP. (**E**) HEK293T cells transfected with expression vectors for MYB-2KR and p300 were treated for 16 h with Bcr-TMP, as indicated at the bottom. Total cell extracts were then analyzed by Western blotting for expression of p300, MYB-2KR, acetylated p300, acetylated MYB-2KR, and β-actin. (**F**) HEK293T cells were transiently transfected with the C/EBP-dependent reporter plasmid pmim3mim-Luc and expression vectors for human C/EBPβ and p300, as indicated. Transfected cells were treated with Bcr-TMP for 16 h and subjected to luciferase assays. The columns show the absolute luciferase values in arbitrary numbers. Asterisks in panels B and F indicate statistical significance (ns: non-specific; ** *p* < 0.01; *** *p* < 0.001; Student’s *t*-test). The uncropped Western blots have been shown in Figure S2.

As a first step to explore the inhibitory mechanism of Bcr-TMP we studied its effect on the DNA-binding activity of MYB. Nuclear extracts from HEK293T cells expressing MYB-CT3 and treated for 16 h with or without Bcr-TMP were used to perform EMSA. Incubation of a radiolabeled oligonucleotide containing a MYB-binding site with nuclear extract showed several complexes with retarded mobility, two of which (black dots in Figure 2A) were MYB-specific as they only appeared with extracts from cells expressing MYB-CT3. We observed no decrease in these complexes at compound concentrations that strongly reduced the activity of MYB-CT3 (Figure 2A). Additional EMSA experiments with nuclear extracts from MYB-CT3 expressing cells treated with significantly higher compound concentrations in vitro showed no decrease in the MYB-specific complexes (right part of Figure 2A). We conclude that Bcr-TMP does not interfere with MYB DNA-binding activity.

Because the sumoylation-deficient MYB mutant MYB-2KR recruits co-activator p300 more efficiently than wild-type MYB [47], the finding that MYB-2KR was more sensitive to Bcr-TMP than wild-type MYB (Figure 1C) pointed to a potential role of p300 in the inhibitory mechanism. To investigate if Bcr-TMP affects the cooperation of MYB with p300, we performed reporter assays with HEK293T cells expressing MYB-2KR in the absence or presence of p300. Although the activity of MYB-2KR was substantially increased by p300 the inhibition mediated by the compound was very similar in the presence or absence of ectopic p300, as demonstrated by normalizing the luciferase values in the absence of the compound (Figure 2B). This suggests that Bcr-TMP interferes with the ability of p300 to stimulate MYB activity.

The MYB point mutant M303V decreases MYB activity due to reduced interaction with p300 [16,17,18], thereby mimicking a decrease in the endogenous p300 expression. Comparison of the effects of Bcr-TMP on wt and M303V MYB provided a further possibility to investigate whether or not the compound mainly acts by inhibiting the cooperation of MYB with p300. In case the compound inhibited MYB activity via a p300-independent cooperation partner, we expected its inhibitory effect on the M303V mutant to be stronger than on wild-type MYB, because the relative contribution of the p300-independent cooperation partner to the residual activity of the M303V mutant would be higher than in case of wild-type MYB. Figure 2C shows the effects of Bcr-TMP on wild-type and M303V MYB-CT3. The left panel shows that the M303V mutation had approximately only 30% residual activity, confirming the effect of the mutation. To compare the inhibition caused by Bcr-TMP on both versions of MYB-CT3, their activities are presented after normalizing the activity in the absence of inhibitor to 100% (Figure 2C). This showed that the extent of inhibition at each tested concentration of Bcr-TMP was identical for wild-type and mutant MYB, supporting the notion that the compound suppresses the activity of MYB in a p300-dependent manner.

To address how Bcr-TMP affects the cooperation of MYB and p300 we first asked if the compound down-regulates the expression of endogenous p300. Figure 2D shows that this was not the case. Acetylation of specific lysine residues in the C-terminal part of MYB by p300 had been shown to stimulate MYB activity [9,51]. We considered acetylation of MYB unlikely to play a major role in the inhibitory mechanism because the C-terminally truncated MYB-CT3 is clearly inhibited although lacks these acetylation sites. Nevertheless, we examined if Bcr-TMP affects the acetylation of MYB or of p300 itself. However, p300-dependent acetylation of MYB and auto-acetylation of p300 were un-affected by the compound (Figure 2E). Since acetylation of MYB requires interaction with p300 this also excludes that the compound interferes with the MYB-p300 interaction.

That Bcr-TMP disrupts the function of p300 as a MYB co-activator raised the question of whether other transcription factors that recruit p300 are also inhibited by the compound. As a first step to address this question we examined its effect on the activity of C/EBPβ, a transcription factor known to bind to the Taz2 domain of p300 [52]. Figure 2F shows that the stimulation of the activity of C/EBPβ by p300 was strongly decreased by the inhibitor. Thus, we conclude that the compound acts as a p300 inhibitor rather than as a MYB inhibitor. However, the inhibitory mechanism of the compound remains to be explored.

### 3.3. Bcr-TMP Induces the Expression of Myeloid Differentiation Markers and Cell Death in AML Cell Lines

**Figure 3:**
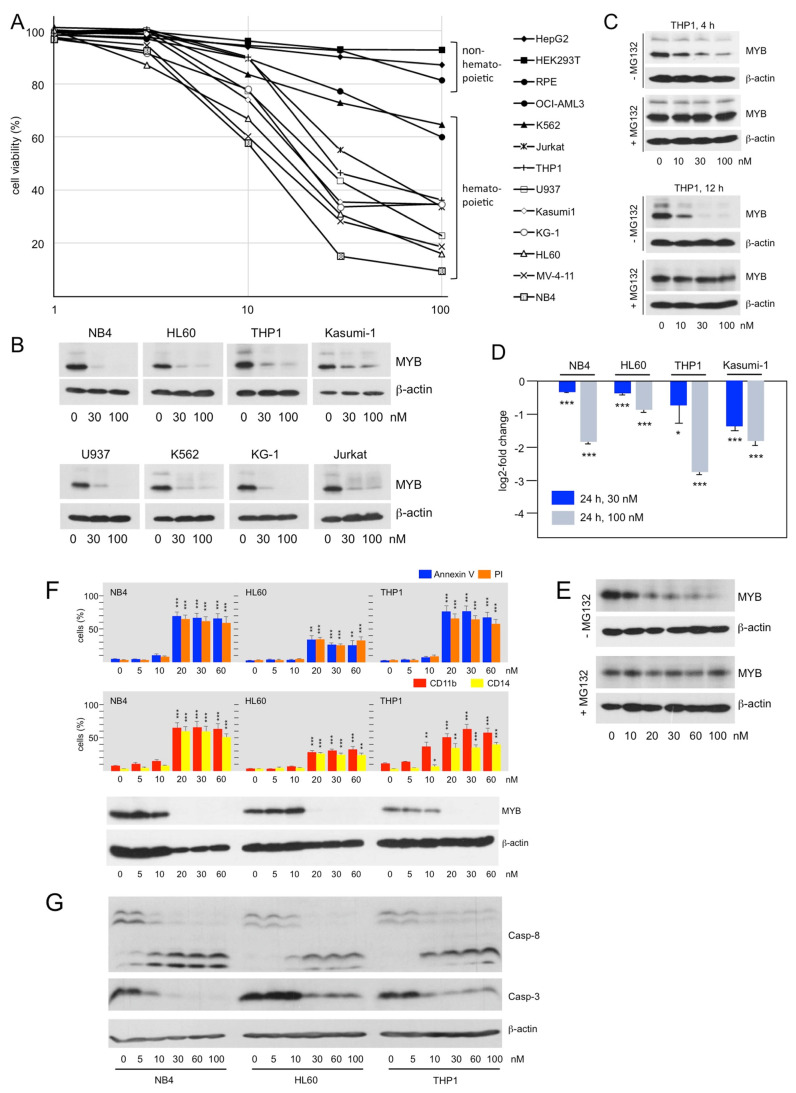
Bcr-TMP suppresses viability and MYB expression and induces cell death and differentiation in AML cell lines: (**A**). Inhibition of cell viability by Bcr-TMP. Non-hematopoietic cell lines (HEKT, HepG2, and hTERT-RPE) and MYB-expressing hematopoietic cell lines (HL60, THP1, NB4, OCI-AML3, K562, Jurkat, U937, Kasumi1, KG-1, MV-4-11) were treated for 24 h with the indicated concentrations of Bcr-TMP and analyzed by a MTT assay. The figure shows percent viable cells relative to untreated cells. (**B**) Western blot analysis MYB expression in hematopoietic cell lines treated for 24 h with Bcr-TMP. (**C**) Western blot analysis of MYB expression in THP1 cells treated for 4 or 12 h with Bcr-TMP in the absence or presence of proteasome inhibitor MG132. (**D**) RT-PCR analysis of MYB mRNA expression in selected cell lines treated for 24 h with Bcr-TMP. (**E**) Western blot showing MYB and β-actin expression in MCF7 cells treated for 12 h with the indicated concentrations of Bcr-TMP in the absence or presence of MG132. (**F**) NB4, HL60 and THP1 cells treated for 48 h with Bcr-TMP were stained with FITC-annexin V and propidium iodide (top) or with antibodies against CD11b and CD14 (middle) and analyzed with a flow cytometer. Western blots (bottom) show expression of MYB and β-actin. (**G**) Western blots showing the effect of Bcr-TMP on caspase 3 and 8 expression. Asterisks in panels D and F indicate statistical significance (* *p* < 0.05; ** *p* < 0.01; *** *p* < 0.001; Student’s *t*-test). The uncropped Western blots have been shown in Figure S3.

To investigate the activity of Bcr-TMP in a setting relevant to the oncogenic potential of MYB we examined its effect on human AML cell lines. Figure 3A shows that Bcr-TMP suppressed the viability of MYB-expressing hematopoietic cell lines more potently than that of MYB-negative non-hematopoietic cell lines, consistent with its MYB inhibitory activity. Western blotting showed that MYB expression was strongly decreased already at a compound concentration of 30 nM in all hematopoietic cell lines analyzed (Figure 3B). We used THP1 cells to demonstrate that this decrease was abolished by the proteasome inhibitor MG132, indicating that it was mainly due to proteasomal degradation (Figure 3C). Comparison of MYB expression after treatment with the compound for 4 and 12 h showed that proteasomal degradation was a slow process that occurred over several hours. Furthermore, MYB mRNA expression was also diminished, as shown by RT-PCR analysis of RNA from cells treated for 24 h with the inhibitor (Figure 3D). Since MYB expression was not decreased in HEK293-derived screening cells we wondered whether Bcr-TMP-induced degradation of MYB was specific to hematopoietic cells. We used the MYB-positive breast cancer cell line MCF7 and found that Bcr-TMP also caused degradation of MYB in these cells (Figure 3E).

FITC-annexin V and propidium iodide staining of NB4, HL60, and THP1 cells treated for 48 h with different concentrations of Bcr-TMP and subsequent analysis by flow-cytometry showed that the compound induced apoptotic and necrotic cell death at concentrations of 20 nM or above. The compound also induced expression of the myeloid-differentiation markers CD11b and CD14 (Figure 3F). The induction of apoptosis was further confirmed by demonstrating proteolytic processing of caspases 3 and 8 (Figure 3G).

**Figure 4:**
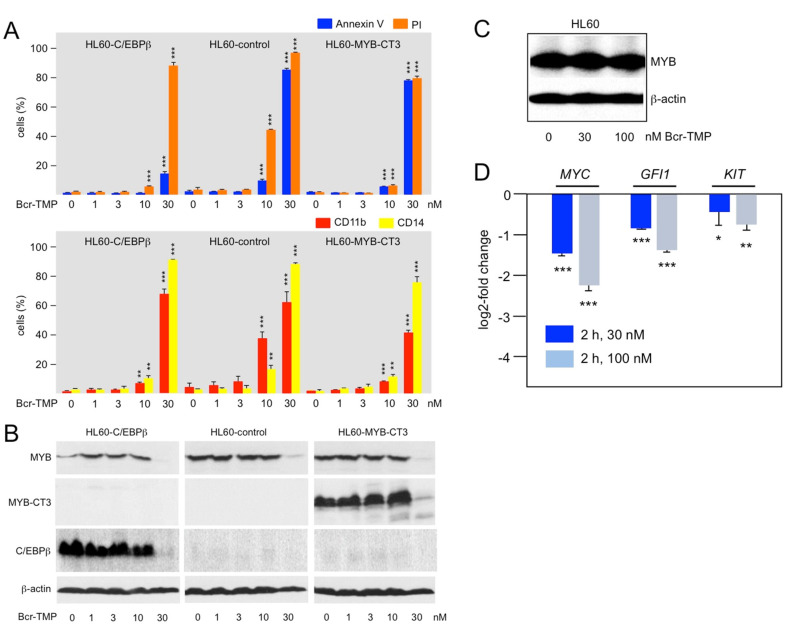
Bcr-TMP suppresses AML cells in a MYB-dependent manner: (**A**) HL60 cells infected with a control lentivirus (HL60-control) or a lentivirus encoding C-terminally truncated MYB (HL60-CT3) or C/EBPβ (HL60-C/EBPβ) were treated for 72 h with the indicated concentrations of Bcr-TMP, followed by staining with annexin V and propidium iodide (top) or antibodies against CD11b and CD14 (bottom) and analysis by flow cytometry. (**B**) Western blot analysis of MYB and C/EBPβ expression of the cells treated as in panel A. (**C**,**D**) HL60 cells treated for 2 h with 30 or 100 nM Bcr-TMP. The cells were the analyzed by Western blotting for MYB and β-actin expression (**C**) and by RT-PCR analysis for *MYC*, *GFI1* and *KIT* mRNA expression (**D**). Asterisks indicate statistical significance (* *p* < 0.05; ** *p* < 0.01, *** *p* < 0.001, Student’s *t*-test). The uncropped Western blots have been shown in Figure S4.

To investigate if the ability to inhibit the MYB transactivation potential also contributes to the effects of Bcr-TMP elicited in AML cells we employed HL60 cells overexpressing MYB-CT3, a C-terminally truncated MYB that is more active than wild-type MYB [53]. HL60-control cells (expressing only endogenous MYB) and HL60 cells expressing MYB-CT3 were treated for 72 h with Bcr-TMP, using also concentrations that were too low to induce degradation of MYB. We then analyzed the cells for apoptotic and necrotic cell death and expression of the myeloid differentiation markers CD11b and CD14. While control HL60 cells showed increased cell death and differentiation at 10 nM of Bcr-TMP, MYB-CT3 expression largely rescued the cells from these effects of Bcr-TMP (Figure 4A, middle and right panel). As demonstrated by Western blotting, there was no decrease in endogenous MYB or ectopic MYB-CT3 expression at this inhibitor concentration (Figure 4B). By contrast, at 30 nM inhibitor concentration, the expression of endogenous MYB and ectopic MYB-CT3 was strongly diminished and the rescueing effect of MYB-CT3 was lost. This was accompanied by increased expression of cell-death and differentiation markers in both cell lines. Overall, these data show that expression of the activated MYB-CT3 counteracts the activity of the compound to a significant extent and indicate that Bcr-TMP exerts these effects not only by inducing MYB degradation but also by inhibiting MYB activity.

To further support the notion that Bcr-TMP inhibits MYB activity independently of inducing its degradation we considered that degradation of MYB is a relatively slow process. Thus, we treated HL60 cells for only 2 h with the compound and MYB expression was not reduced under these conditions (Figure 4C). At the same time, RT-PCR analysis showed that the mRNA expression of the direct MYB target genes *MYC*, *GFI1*, and *KIT* was significantly decreased (Figure 4D). The short treatment time made it likely that the down-regulation of these genes was due to direct effects on MYB activity rather than caused indirectly by changes in the differentiation state of the cells. Overall, these data support the notion that Bcr-TMP decreases MYB activity in AML cells and confirm that inhibition of MYB activity and induction of its degradation are distinct activities of Bcr-TMP that differ in concentration- and time-dependent manner.

### 3.4. Bcr-TMP Targets a MYB-C/EBPβ-p300 Transcriptional Module

We have previously observed that small-molecule mediated inhibition of transcription factor C/EBPβ exerted similar effects on MYB-regulated genes and caused comparable biological effects in AML cells as inhibition of MYB itself [37,42,45]. This led us to propose that MYB acts in concert with C/EBPβ and p300 to form a “transcriptional module” that controls the expression of genes involved in maintaining AML cells in an undifferentiated state [45]. Support for this idea came from the analysis of the *GFI1* gene, whose expression is required for the maintenance of hematopoietic stem and progenitor cells [54,55,56,57]. We showed that *GFI1* expression is regulated in a direct manner via MYB and C/EBP binding sites in the *GFI1* promoter through the cooperation of MYB, C/EBPβ, and p300 [45]. To examine if Bcr-TMP disrupts the function of this module in AML cells we compared the effect of Bcr-TMP on HL60-control cells and HL60 cells expressing ectopic C/EBPβ. Treating the cells with 10 nM Bcr-TMP largely rescued the C/EBPβ expressing cells from inhibitor-induced cell death and expression of myeloid differentiation markers, similar to expression of MYB-CT3 (middle and left panel of Figure 4A). As in case of ectopic MYB-CT3 expression, C/EBPβ expression was strongly reduced at 30 nM inhibitor concentration and the protective effect of C/EBPβ was largely lost. Overall, these data further support the idea of MYB, C/EBPβ, and p300 forming the core of a transcriptional module in myeloid cells whose activity is disrupted by Bcr-TMP.

### 3.5. Bcr-TMP Suppresses the Proliferation of Murine MLL-AF9 Transformed AML Cells and of Patient-Derived ACC Cells

**Figure 5:**
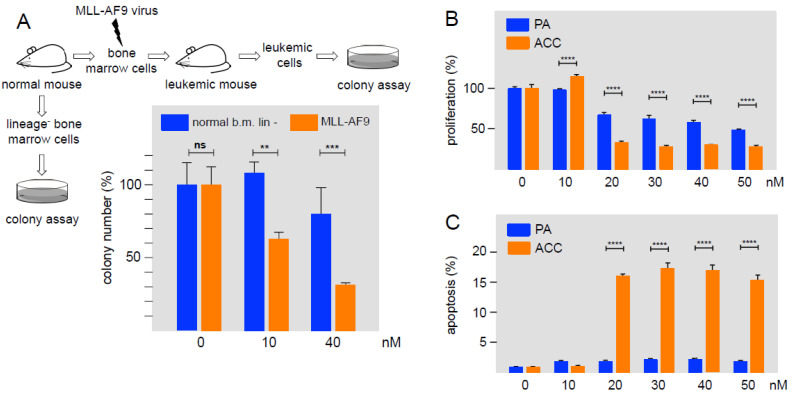
Effect of Bcr-TMP on primary murine AML cells and patient-derived human ACC cells. (**A**) Murine lineage-negative (lin-) cells from the bone marrow of healthy mice and murine *MLL/AF9*-transformed AML progenitor cells were subjected to colony-formation unit assays in the absence or presence of Bcr-TMP. Equal cell numbers were plated with DMSO or with Bcr-TMP at the indicated concentrations. Columns show the relative colony number and standard deviation, normalized to the colony number of the DMSO control. Asterisks indicate statistical significance (ns: non-specific; ** *p* < 0.01, *** *p* < 0.001, Student’s *t*-test). (**B**) Cell viability assay of patient-derived ACC cells and primary pleomorphic adenoma cells (PA), treated with the indicated concentrations of Bcr-TMP for 72 h. (**C**) Induction of apoptosis by Bcr-TMP in ACC and PA cells. Cells were treated for 24 h with the indicated compound concentrations. Asterisks indicate statistically significant differences between ACC and control cells for each concentration (*** *p <* 0.001; **** *p <* 0.0001; one-way ANOVA).

As a first step to examine the activity of Bcr-TMP in a disease-relevant context, we tested its effect on MLL-AF9 transformed primary murine AML cells and human ACC cells. Previous work had shown that AML cells are more vulnerable to MYB inhibition than their normal counterparts [26,37]. It was therefore interesting to see if Bcr-TMP also affects the proliferation of primary AML cells and normal hematopoietic progenitor cells differentially. We prepared murine *MLL-AF9* transformed AML stem cells from leukemic mice and lineage-negative early hematopoietic progenitors isolated from the bone marrow of healthy mice, and subjected them to colony-forming assays in the presence or absence of Bcr-TMP. Figure 5A shows that the clonal proliferation of *MLL-AF9* transformed cells was suppressed by the compound significantly stronger than the proliferation of normal progenitor cells, consistent with the addiction of AML cells to high levels of MYB activity.

As a second approach we used patient-derived ACC cells that represents a second type of MYB-dependent neoplasm that is associated with the expression of oncogenic MYB-NFIB fusion proteins due to *MYB* and *NFIB* gene fusions. Figure 5B shows that the proliferation of ACC cells was suppressed at inhibitor concentrations of 20 nM or higher. As control, non-malignant pleomorphic adenoma (PA) cells were significantly less sensitive to the compound. The same concentrations of the compound also induced apoptosis in ACC cells more strongly than in PA cells (Figure 5C). Overall, these results show that cells derived from two different MYB-dependent malignancies are highly sensitive to nanomolar concentrations of Bcr-TMP.

### 3.6. Bcr-TMP Exhibits Microtubule-Destabilizing Activity

**Figure 6:**
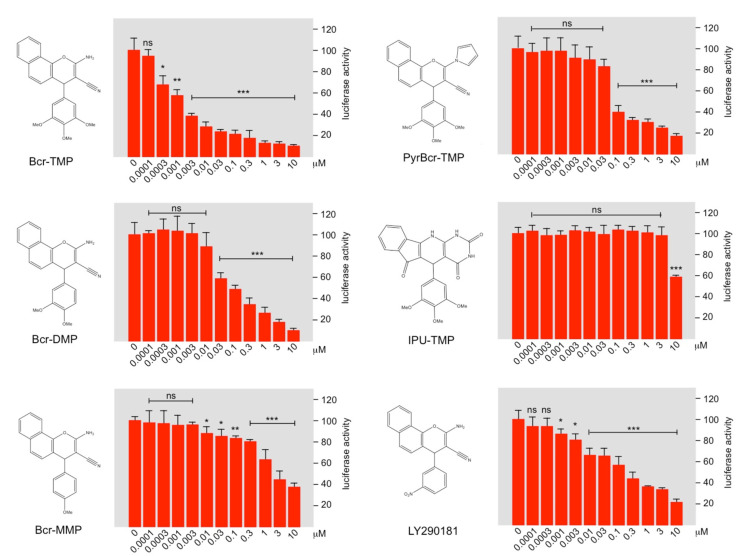
Structure–activity analysis of Bcr-TMP-related compounds. The columns in each panel show the relative luciferase activity in HEKT cells transfected with reporter plasmid pGL4-5xMRE(GG)-Myc and expression vector for MYB-2KR, treated for 16 h with the indicated concentrations of the compounds shown to the left. Asterisks indicate statistical significance (* *p* < 0.05; ** *p* < 0.01, *** *p* < 0.001, Student’s *t*-test).

**Figure 7:**
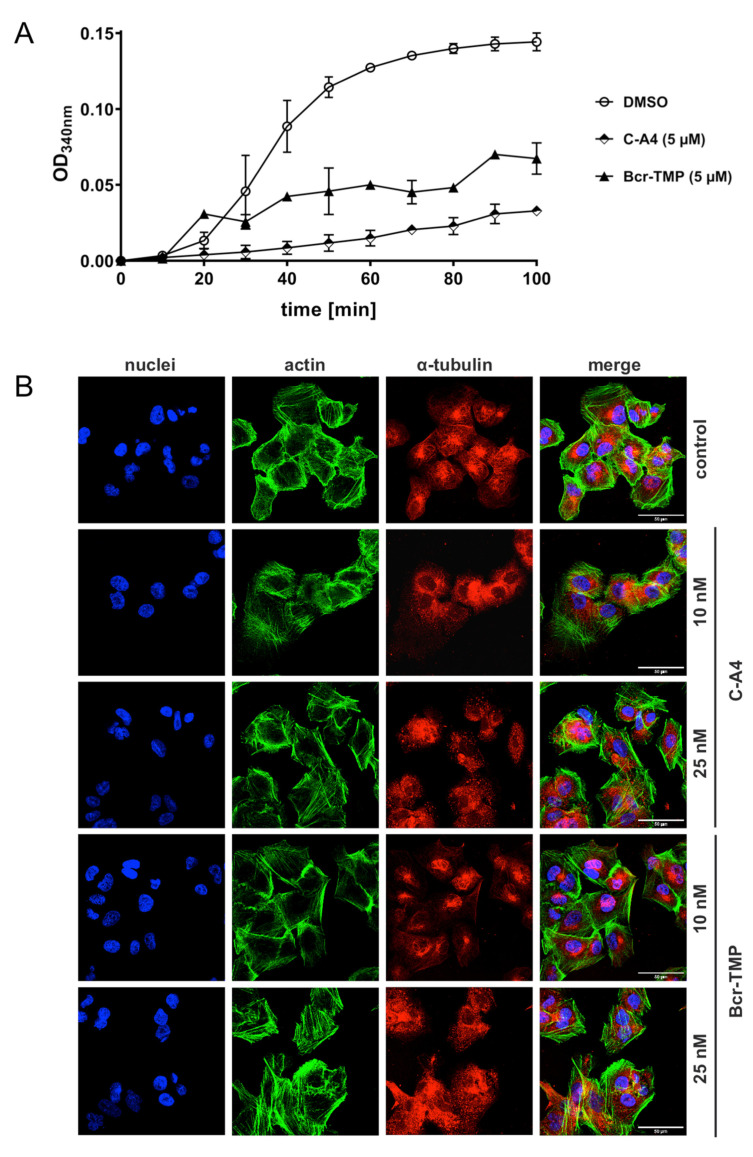
Bcr-TMP inhibits tubulin polymerization and disrupts the microtubule network in 518A2 melanoma cells: (**A**) Effect of Bcr-TMP and combretastatin A4 (5 μM each) on tubulin polymerization in a turbidimetric in vitro tubulin polymerization assay. DMSO served as negative control. (**B**) Concentration dependent effect of 10 and 25 nM combretastatin A4 or Bcr-TMP on the tubulin and actin cytoskeleton of 518A2 melanoma cells after 24 h of treatment. DMSO treated cells served as negative control. Individual panels show nuclei (blue), actin (green), and tubulin (red) as well as a merge of all three. Images are representative of at least three independent experiments. The scale bar (bottom right in the leftmost panels) corresponds to 50 μm.

As a first step towards investigating structure–activity relationships of Bcr-TMP we compared the MYB-inhibitory activities of several related compounds, using HEK293T cells transfected with the MYB-responsive luciferase reporter plasmid and an expression vector for MYB-2KR. As illustrated in Figure 6, changes in the trimethoxy-phenyl or benzochromene groups decreased the inhibitory activity, indicating that both moieties contribute to the activity of Bcr-TMP. Interestingly, the related compound LY290181, which has previously been implicated in microtubule destabilization due to its ability to bind to tubulin [58,59], also had significant MYB-inhibitory activity. This alerted us to the possibility that Bcr-TMP might also have microtubule-destabilizing activity. To address this, we compared the effects of Bcr-TMP and combretastatin A4, a known tubulin-binding agent [60], in an in vitro tubulin polymerization assay. Interestingly, Bcr-TMP interfered with tubulin polymerization in this assay somewhat less potently than combretastatin A4 (Figure 7A). Microscopic examination of the microtubule organization in cells treated with Bcr-TMP or combretastatin A4 clearly showed that the microtubule cytoskeleton was disrupted by both compounds (Figure 7B). Consistent with the in vitro polymerization assay, combretastatin had a slightly stronger effect on the microtubule organization than Bcr-TMP (compare the effects of 10 nM concentration of both compounds). Overall, these results indicate that Bcr-TMP is a dual-active compound that inhibits MYB activity and microtubule organization and raise the possibility of a previously unknown link between microtubule-disruption and inhibition of MYB activity.

## 4. Discussion

MYB is a master transcription regulator of hematopoietic cells that has gained attention as a potential drug target for AML and ACC [34,35,39]. Previous studies have indicated that MYB is a druggable transcription factor and suggested that strategies based on inhibition of MYB may open new therapeutic avenues for the treatment of neoplasms depending on deregulated MYB [36,37,38,39,40,41,42]. In an attempt to expand the spectrum of potential MYB inhibitors we have discovered Bcr-TMP as a nanomolar-active MYB-inhibitory compound, which was described before as a potent caspase activator in human breast and non-small-cell lung cancer cells [61]. In AML cells, Bcr-TMP induces apoptotic and necrotic cell death, expression of myeloid differentiation markers, and down-regulation of direct MYB target genes, as exemplified by *MYC*, *GFI1*, and *KIT*. In colony assays, the compound affects MYB-addicted primary murine AML cells more strongly than non-malignant hematopoietic progenitor cells. Moreover, the compound also exerts pronounced anti-proliferative effects on MYB-NFIB positive ACC cancer cells while related benign PA cells are affected significantly less. Taken together, this makes Bcr-TMP an interesting novel MYB-inhibitory agent that warrants further studies of its therapeutic potential and its mechanism of action.

Bcr-TMP affects MYB in two distinct ways, namely, by inducing degradation of MYB and suppressing its transactivation potential. Degradation appears to require a threshold concentration (approximately 30 nM in HL60 cells) and occurs in a concentration-dependent and time-delayed manner. MYB degradation in HL60 and MCF7 cells was blocked by the proteasome inhibitor MG132, suggesting that it proceeds via the ubiquitin–proteasome system. That Bcr-TMP also suppresses the transactivation potential of MYB was initially demonstrated in HEK293T cells, in which degradation of MYB is blocked, presumably due to the expression of the adenoviral E1A protein in these cells. E1A is known to inhibit the degradation of MYB by sequestering FBW7, an F-box protein that recruits MYB among several other proteins to the corresponding ubiquitin ligase complex to facilitate its degradation [62,63]. Suppression of MYB activity by Bcr-TMP is also evident in AML cells at compound concentrations that are not sufficient to induce degradation of MYB. This is seen in Figure 4A, which showed a significant induction of cell-death and differentiation markers by the compound without an apparent decrease in MYB expression. Moreover, these effects were largely suppressed by ectopic expression of an activated version of MYB, confirming that the compound exerts these effects in a MYB-dependent manner. That Bcr-TMP inhibits MYB activity in AML cells is further supported by the down-regulation of several direct MYB target genes already after two hours of treatment, while MYB expression itself was not decreased due to the short incubation time.

Initial mechanistic studies showed that Bcr-TMP interferes with the ability of co-activator p300 to stimulate the activity of MYB, suggesting that Bcr-TMP is not a direct MYB inhibitor but rather a p300 inhibitor. How Bcr-TMP exerts its inhibitory effect on a mechanistic level could unfortunately not be clarified and thus remains to be elucidated. Our studies showed that the compound neither decreased endogenous or ectopic p300 expression, nor did it affect the ability of p300 to acetylate MYB via its histone acetyl-transferase activity. That Bcr-TMP targets p300 is also supported by its inhibitory effect on the stimulation of C/EBPβ activity by p300.

We have previously observed that compounds that inhibit the activity of C/EBPβ, but not of MYB, also down-regulate the expression of many MYB target genes and exert similar biological effects in AML cells as MYB inhibitors [45]. Therefore, we have proposed that MYB, C/EBPβ, and p300 constitute a transcriptional module as an essential part of the oncogenic transcriptional program that is responsible for the maintenance of AML cells in an undifferentiated state [45,64]. This is strongly supported by genome-wide ChIP studies showing that MYB, C/EBPβ, and p300 (together with other myeloid transcription factors) co-localize at many genomic sites in AML cells which correspond to cis-acting transcriptional control regions of genes, such as *MYC* and *GFI1*, which are expressed in these cells [65]. Disruption of this module by inhibition of MYB, C/EBPβ, or p300, as in the case of Bcr-TMP, induces cell death and differentiation. Although p300 acts as a co-activator for many transcription factors [66], we presume that the addiction of AML cells to high levels of MYB activity as well as the synergistic interplay of MYB and C/EBPβ and their simultaneous inhibition via p300 may explain why the compound preferentially inhibits AML cells versus normal hematopoietic progenitor cells or non-hematopoietic cells.

One of the most surprising findings of our study is that Bcr-TMP exhibits an additional activity as microtubule destabilizing agent. This was demonstrated by its ability to inhibit tubulin polymerization in an in vitro assay as well as by microscopic examination of its impact on the cellular microtubule network (Figure 7). Comparison of the chemical structures of Bcr-TMP and combretastatin A4 shows some similarities, such as a trimethoxyphenyl moiety, which is present in both compounds and may have a role in their microtubule-destabilizing activity. The dual activity of Bcr-TMP as a MYB and microtubule inhibitor raises several interesting questions. Are these activities independent of each other or is there a causal link between microtubule destabilization and inhibition of MYB activity? Will the synthesis of chemical variants of Bcr-TMP allow to further dissect MYB-inhibitory and microtubule-destabilizing properties of the compound and eventually to tune both activities relative to each other? Given the emerging concept of polypharmacology, and the growing interest in multitarget drugs as therapeutic agents, it will be interesting to know if MYB inhibitory and anti-microtubule activities both contribute to the ability of the compound to preferentially suppress the proliferation of AML and ACC cells.

## 5. Conclusions

Bcr-TMP is a novel, nanomolar-active multifunctional compound that induces proteasomal degradation of MYB and inhibits its transactivation potential by targeting the co-activation function of p300. The compound displays anti-proliferative activities in human AML cell lines and inhibits MYB-addicted primary murine AML cells and patient-derived ACC cells more potently than their non-oncogenic counterparts. Finally, the compound displays an additional activity as a microtubule-disrupting agent. Further work addressing open questions regarding mechanistic aspects of its p300 inhibitory function, and the possible existence of a link between microtubule-disruption and MYB activity, will be highly interesting.

## Data Availability

No new data were created or analyzed in this study. Data sharing is not applicable to this article.

## Supplementary Materials

The following are available online at https://www.mdpi.com/article/10.3390/cancers14010043/s1, Figure S1: Uncropped Western blots for Figure 1B,C, Figure S2: Uncropped Western blots for Figure 2A,E,D, Figure S3: Uncropped Western blots for Figure 3B,C,E,F,G, Figure S4: Uncropped Western blots for Figure 4B,C.
