# Supplementary material for: Bcr-TMP, a Novel Nanomolar-Active Compound That Exhibits Both MYB- and Microtubule-Inhibitory Activity
Source: Cancers (Basel). 2021 Dec 23;14(1):43. doi: 10.3390/cancers14010043 (PMC8750090; doi:10.3390/cancers14010043)
Supplement: Supplementary file 1 [file cancers-14-00043-s001.zip › cancers-1489831-supplementary.pdf]

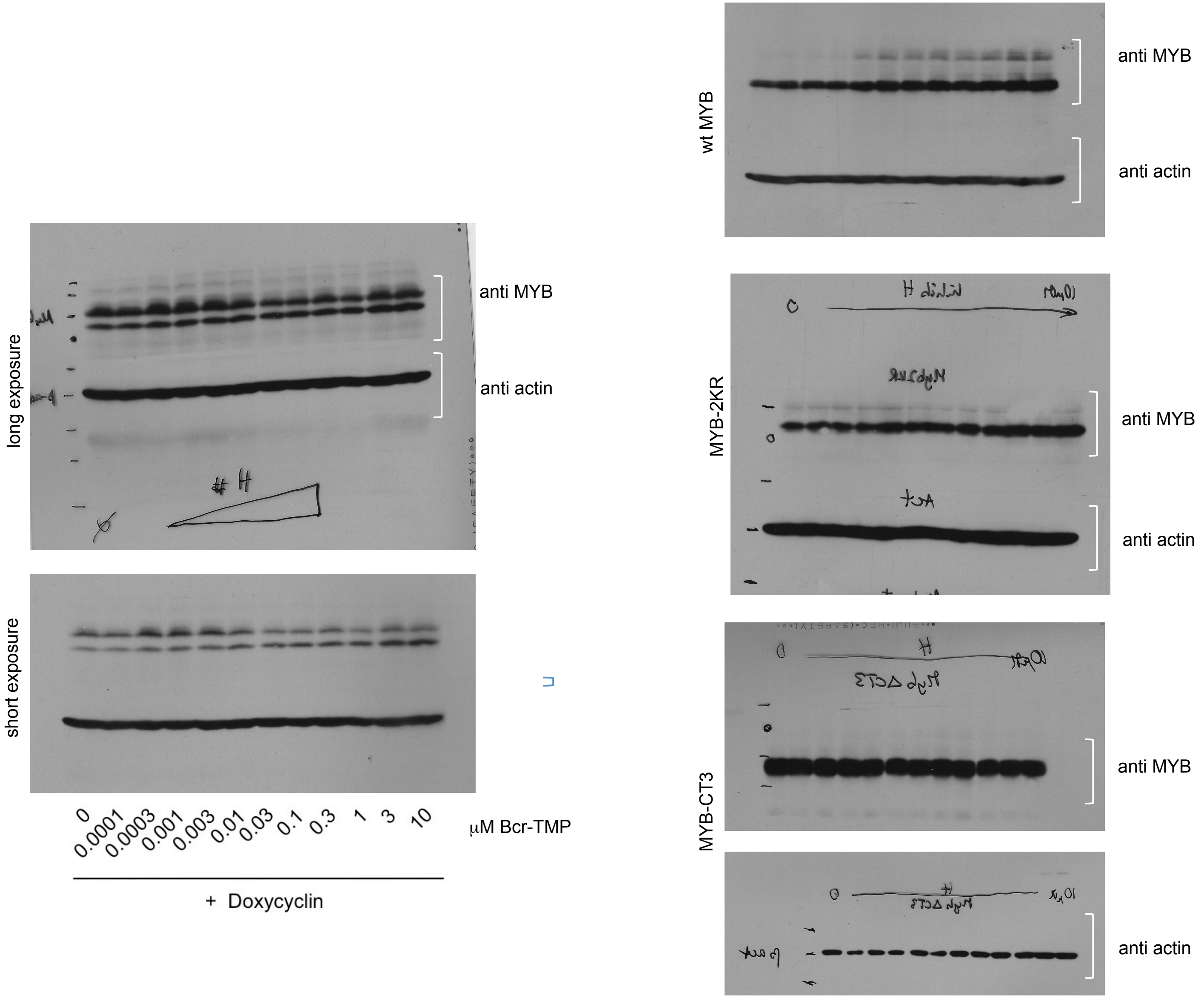

0 μM Bcr-TMP is the first sample on the left,
the last sample on the right is 10 μM in each case

Figure S1. Uncropped Western blots for Figure 1B,C

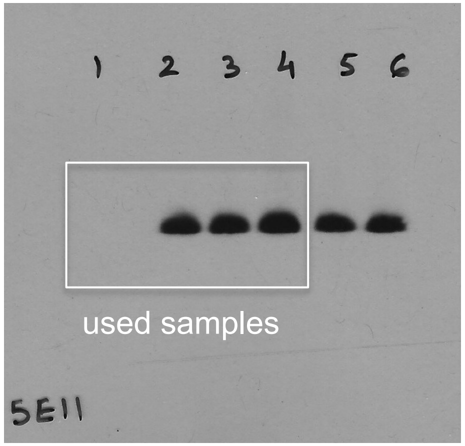

1. Untransfected
2. MYB-CT3 transfected, 0 mM Bcr-MP
3. MYB-CT3 transfected, 1 nM Bcr-MP
4. MYB-CT3 transfected, 30 nM Bcr-MP

5. and 6: irrelevant samples

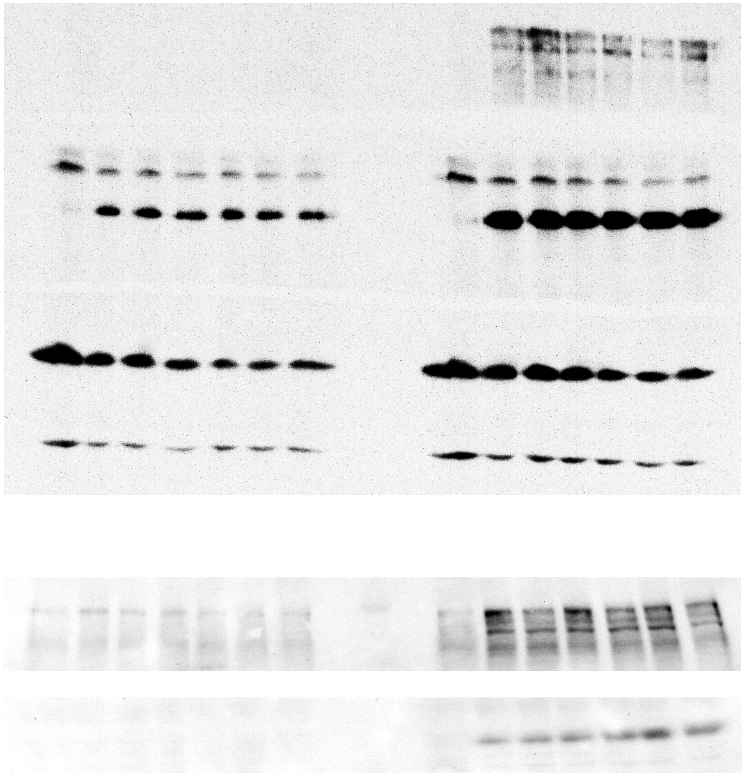

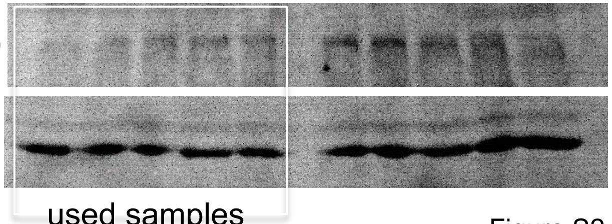

Figure S2. Uncropped Western blots for Figure 2A,E,D

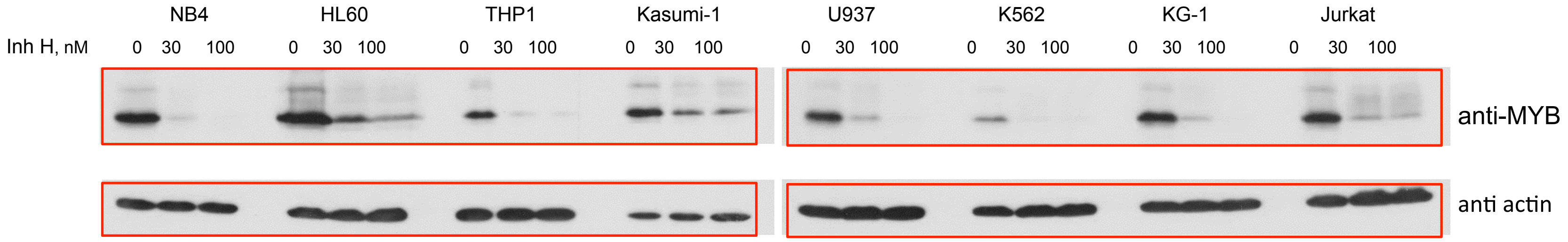

used samples marked by red lines

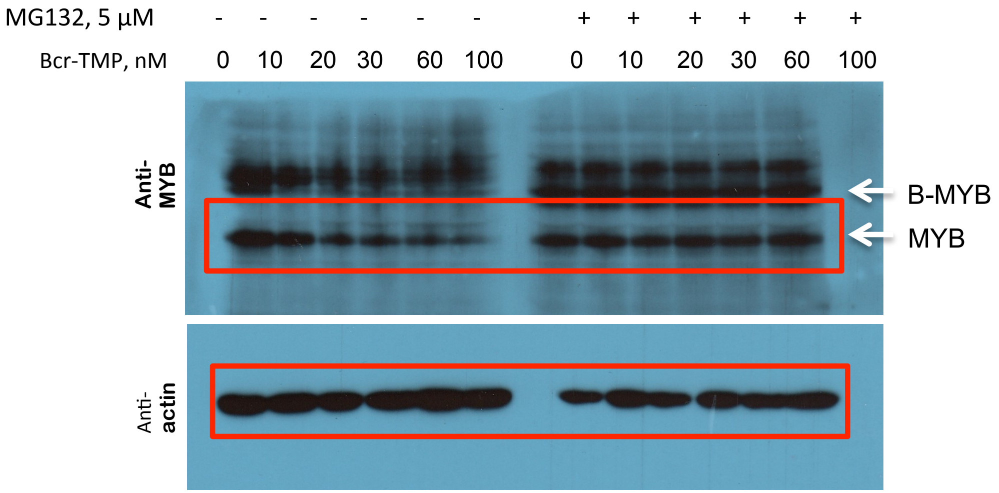

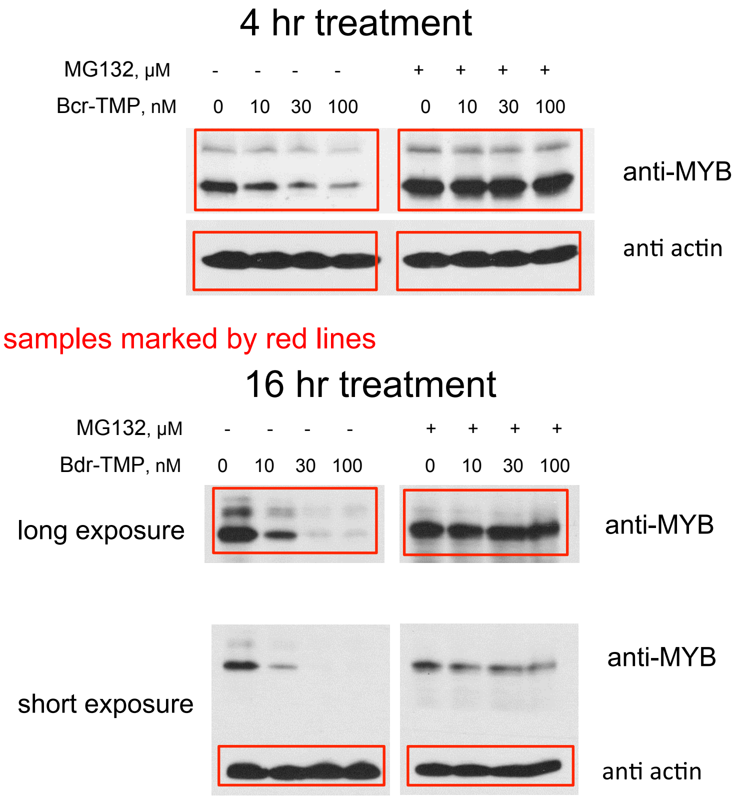

WB 2020-03-31

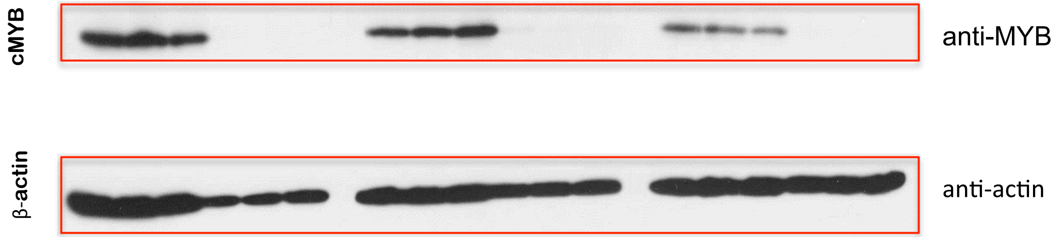

used samples marked by red lines

WB 2020-06-22

24 hours

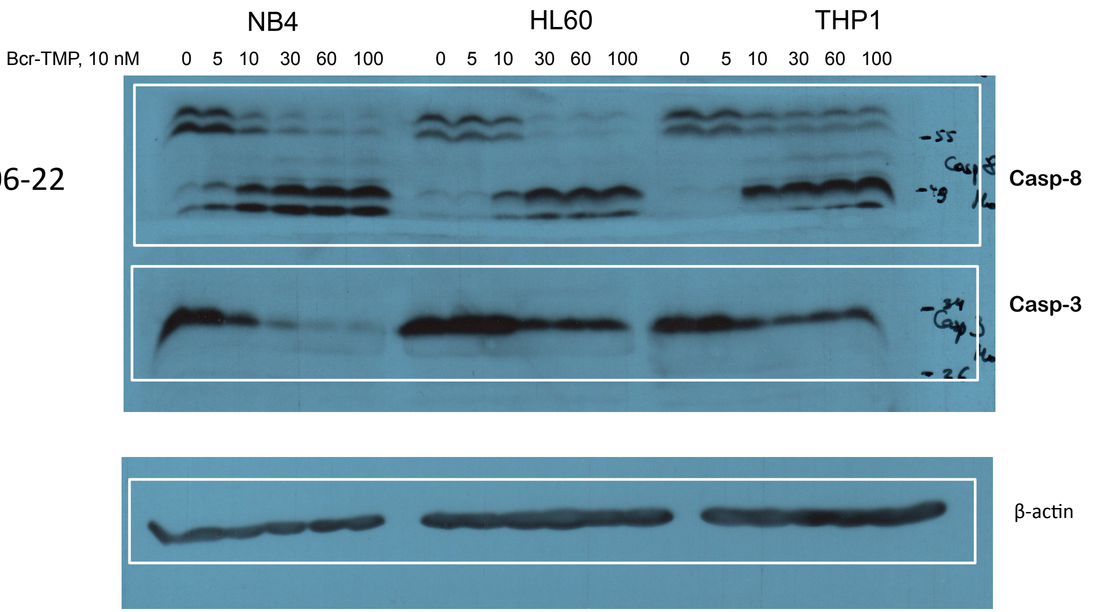

Figure S3. Uncropped Western blots for Figure 3B,C,E,F,G

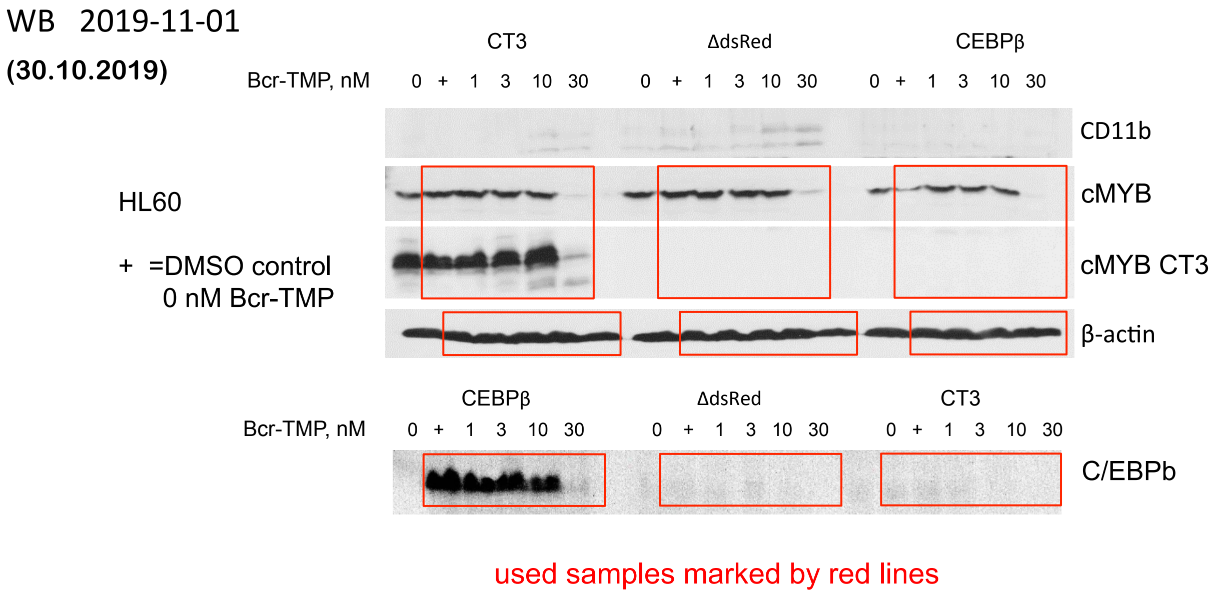

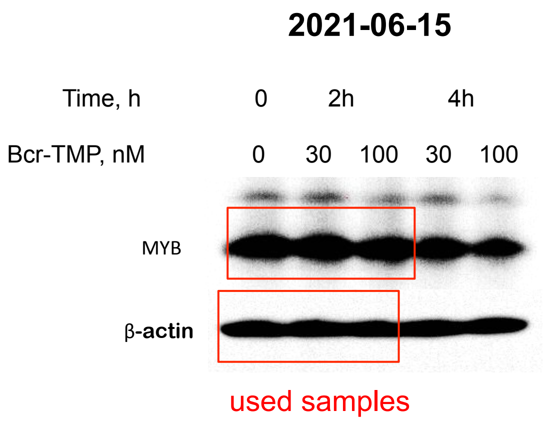

Figure S4. Uncropped Western blots for Figure 4B,C
